# Mathematical topology and geometry-based classification of tauopathies

**DOI:** 10.1038/s41598-024-58221-5

**Published:** 2024-03-30

**Authors:** Masumi Sugiyama, Kenneth S. Kosik, Eleni Panagiotou

**Affiliations:** 1 Department of Mathematics, University of Tennessee at Chattanooga, Chattanooga, TN 37403 USA; 2 Neuroscience Research Institute and Department of Molecular, Cellular, and Developmental Biology, University of California Santa Barbara, Santa Barbara, CA 93106 USA; 3 School of Mathematical and Statistical Sciences, Arizona State University, Tempe, AZ 85281 USA

**Keywords:** Protein folding, Mathematics and computing, Neurological disorders

## Abstract

Neurodegenerative diseases, like Alzheimer’s, are associated with the presence of neurofibrillary lesions formed by tau protein filaments in the cerebral cortex. While it is known that different morphologies of tau filaments characterize different neurodegenerative diseases, there are few metrics of global and local structure complexity that enable to quantify their structural diversity rigorously. In this manuscript, we employ for the first time mathematical topology and geometry to classify neurodegenerative diseases by using cryo-electron microscopy structures of tau filaments that are available in the Protein Data Bank. By employing mathematical topology metrics (Gauss linking integral, writhe and second Vassiliev measure) we achieve a consistent, but more refined classification of tauopathies, than what was previously observed through visual inspection. Our results reveal a hierarchy of classification from global to local topology and geometry characteristics. In particular, we find that tauopathies can be classified with respect to the handedness of their global conformations and the handedness of the relative orientations of their repeats. Progressive supranuclear palsy is identified as an outlier, with a more complex structure than the rest, reflected by a small, but observable knotoid structure (a diagrammatic structure representing non-trivial topology). This topological characteristic can be attributed to a pattern in the beginning of the R3 repeat that is present in all tauopathies but at different extent. Moreover, by comparing single filament to paired filament structures within tauopathies we find a consistent change in the side-chain orientations with respect to the alpha carbon atoms at the area of interaction.

## Introduction

Millions of people worldwide are affected by neurodegenerative diseases which significantly impact their life quality and can lead to death^1^. Such diseases are often characterized by the presence of neurofibrillary lesions in the cerebral cortex which consist of tau protein filaments. Neurodegenerative diseases that are characterized by the deposition of tau protein aggregates are referred to as tauopathies. These include Alzheimer’s disease (AD), primary age-related tauopathy (PART), chronic traumatic encephalopathy (CTE), pick’s disease (PiD), corticobasal degeneration (CBD), argyrophilic grain disease (AGD), progressive supranuclear palsy (PSP), and globular glial tauopathy (GGT). Cryo-electron microscopy (cryo-EM) has enabled the identification of distinct tau protein structures associated to those diseases^2^. The findings by cryo-EM are consistent with the hypothesis that fibril shapes are the determinant of the selectivity of conformation-dependent antibodies, such as GT-7, GT-38, and 18F12 that can distinguish AD-tau from pathological tau of other tauopathies^3,4^. Hence, verifying and understanding the mechanisms of tau fibril shape formation and propagation is crucial for therapeutic development^5,6^. In this manuscript, we employ mathematical topology and geometry to rigorously characterize and classify the shape of different tau structures.

**Figure 1:**
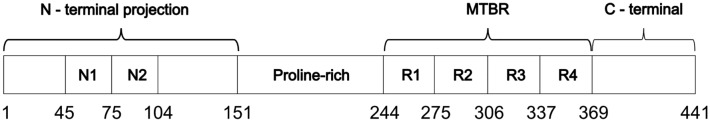
Tau amino acid sequence and regions of the longest 4R tau isoform (2N4R) consisting of 441 amino acids. Six isoforms differ by differential inclusion of *N*1, *N*2, and *R*2. The microtuble-binding repeat region (MTBR) of 4*R* tau isoforms comprise all four repeats (R1—R4) while that of 3*R* tau isoforms are missing *R*2.

**Figure 2:**
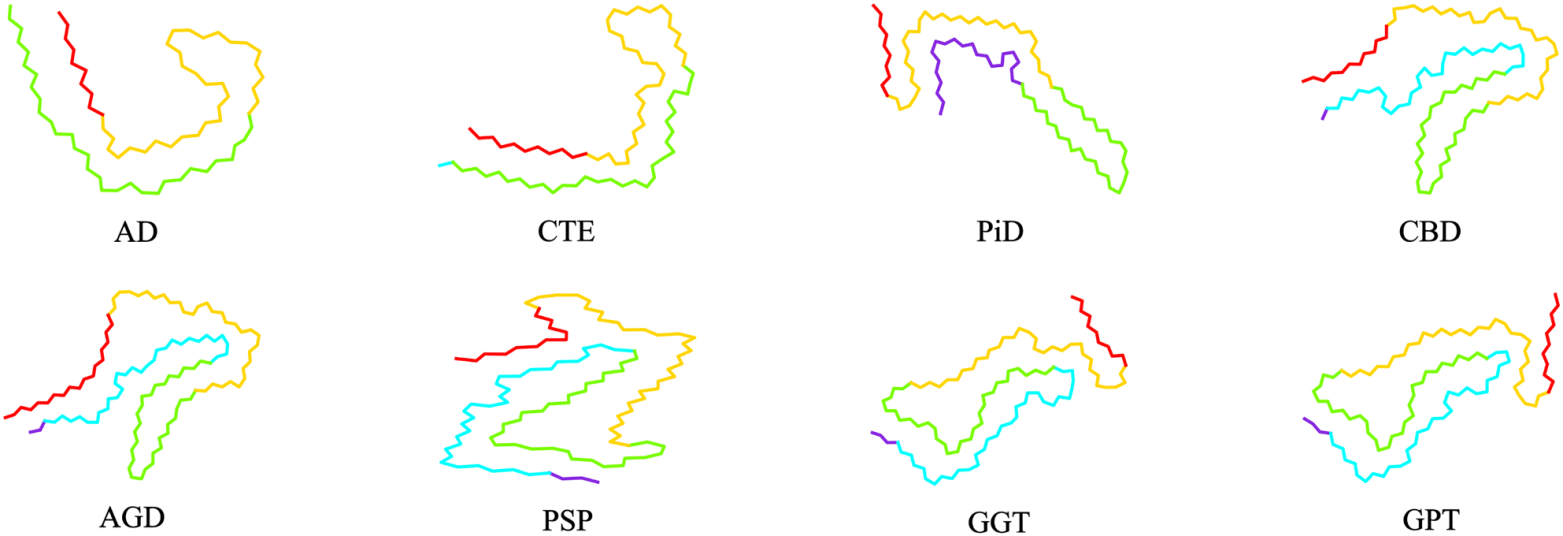
Cartoon representations of different tau folds seen in tauopathies^2,8,9,14,15,30^. Residues in R1–R4 repeats and in the C-terminal region are coloured purple, blue, green, yellow and red, respectively. Each fold consists of the following residues: 306–378 for AD (PDB: *5o3t*), 305–379 for CTE (PDB: *6nwp*), 254–274 and 306–378 for PiD (PDB: *6gx5*), 274–380 for CBD (PDB: *6tjo*), 273–387 for AGD (PDB: *7p6d*), 272–381 for PSP (PDB: *7p65*), 272–379 for GGT (PDB: *7p66*) and GPT (PDB: *7p6a*). The cartoon representations are obtained by projecting the corresponding PDB coordinates to the *xy*-plane.

**Figure 3:**
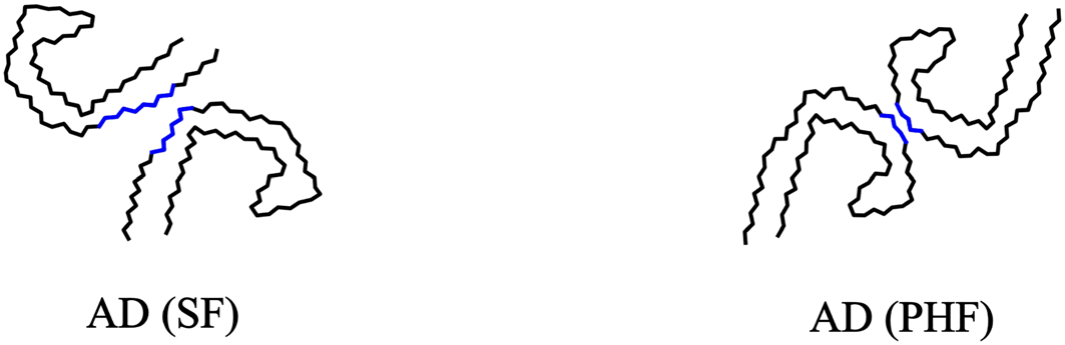
Cartoon representations of two different types of filaments in Alzheimer’s disease are shown. All filaments consist of the residues 306–378. The cartoon representations are obtained by projecting the corresponding PDB coordinates to the *xy*-plane. (Left) the straight filament (SF) (PDB: 5*o*3*t*). The two identical protofilaments are paired back-to-base. (Right) the paired helical filament (PHF) (PDB: 5*o*3*l*). The two identical protofilaments are paired base-to-base. The blue segments indicate the regions in contact in the pairs of filaments; these are within R3 repeat.

Tau protein is an intrinsically disordered protein with a low content of secondary structure and a high level of conformational flexibility^7^. The tau amino acid (residue) sequence can be divided into several regions^8^, see Fig. 1, one of which is the microtubule-binding repeat region (MTBR), formed by repeats R1 to R4 (31 or 32 residues for each repeat and inter-repeat region) that have similar sequences^9^. Under conditions that are not fully understood, tau proteins can undergo a disorder-to-order transition leading to dysfunctional protein behavior, which is promoted by conformational changes and misfoldings that lead to highly ordered tau protein aggregates^10–12^. In Ref.^2^, using cryo-EM it was shown that the folded structures of tau protein filaments for AD, CTE, PiD, CBD, AGD, PSP and GGT are distinct and that some tauopathies such as AD and PART share the same fold. All the distinct tau folds contain the common ordered core which includes R3 and R4 repeats in the MTBR plus an additional 10–13 residues in the C-terminal region. Cartoon representations of tau folds are shown in Fig. 2. The folded structure of tau filaments mainly consist of three or four repeats or both, called 3R, 4R and 3R+4R tauopathies, respectively. Folds observed for 4R tauopathies comprise all of R2 and one or two residues of R1 in addition to the common ordered core. The first level of classification is based on the extent of the common ordered cores, and coincides with the isoform compositions of tau inclusions in the corresponding diseases. At a second level, within 3R+4R tauopathies, the CTE fold is distinct from the AD fold. Also at a second level, 4R tauopathies are divided into two classes on the basis of three-layered and four-layered folds which agrees with observations on post-translational modifications^2,13^. A third level of classification for 4R tauopathies is provided by differences at the residue level between the three- and the four-layered folds. Non-proteinaceous molecule densities have also been associated within the core of tau filament structures^2,14–16^. Some tauopathies consist in different types of spatial organization of filaments present. Namely, two different types of AD and CTE are formed from two identical protofilaments that differ in the interaction between these protofilaments, the straight filament (SF) and the paired helical filaments (PHF) for AD and the type I and type II filaments for CTE, see Fig. 3. The interfaces of these filaments in AD and CTE occur in R3 repeat. The type I and type II filaments in CBD and AGD consist of a single protofilament and a pair of identical protofilaments whose interface involves R4 repeat. For GGT and GPT (a special type of fold that resembles both the PSP and GGT folds), three types of filaments are observed with the type I being composed of a single protofilament and the type II and type III consisting of two identical protofilaments whose interfaces involve both R2 and R4 repeats.

Even though descriptive analyses of structures can be very helpful, protein structure can be rigorously assessed and compared by using mathematical metrics, such as end-to-end distances, or ramachandran plots^5,17,18^. Methods from mathematical topology can provide more accurate measurable characterization of protein structure complexity both locally and globally^19–23^. In particular, methods from knot theory enable the characterization of complexity of both unknotted and knotted proteins^21,23–28^. Recent work has shown a connection between novel topological metrics and protein kinetics, namely, that experimental protein folding rates correlate with the topological structural complexity of the native state of simple, 2-state proteins without knots or slipknots^23^.

In this manuscript, we employ mathematical measures from topology that apply to proteins and to protein fragments. More precisely, we use the linking number, writhe and second Vassiliev measure that provide quantitative topological metrics for global and local classifications of tauopathies based on the structures of tau filaments. The linking number provides the degree of interwinding of filaments around each other while the writhe provides the degree of interwinding of a filament around itself^26^. The second Vassiliev measure provides the degree of higher order complexity of a filament^29^. We find that, eventhough tau filaments are unknotted and unlinked when stacked in aggregates, they have non zero topological signatures for all these metrics that enable to compare and classify them based on subtle quantitative differences. The global structure of filaments gives a classification in terms of the global handedness of their conformation reflected by their writhe; in left-handed (AD, PART, CTE, PiD) and right-handed (CBD, AGD, PSP, GGT, GPT). Notice that this is consistent with PSP, GGT, GPT, AGD and CBD being 4R tauopathies, that distinguishes them from AD and CTE which are 3R+4R tauopathies. Also, at a global level, PSP and GPT (Type Ia and II), appear as outliers, showing a non-trivial higher order complexity, the presence of a knotoid structure. This is reflected by a greater second Vassiliev measure than the other tauopathies. A more refined classification of tauopathies comes from the handedness of the relative interactions between their repeats. These reveal the following sub-clusters: (Ai) AD, PART, CTE, (Aii) PiD, (Bi) CBD, (Bii) AGD, (C) GPT, (Di) PSP, (Dii) GGT. This classification is confirmed by the linking fingerprints of all tauopathies that account for multi-level linking between parts of the filaments, which also reveals many subtle differences among filaments in the same sub-cluster. Our results also reveal that the relative orientation of side-chains and the filament alpha carbon backbone varies among tauopathies. In particular, we find a consistent variation of the side-chain orientations relative to the backbone in single or paired filaments, which is often concentrated in the repeat in contact. These results reveal new aspects of tau filament structure that can be used to rigorously classify tau filament structure and also to create a new mathematical framework for understanding tau protein misfolding and aggregation.

## Results

We represent proteins by their consecutive alpha carbon atoms (CA atoms) as linear open-ended polygonal curves in 3-space, which we use as approximations of the protein backbones. We employ the Gauss linking integral, the writhe and the second Vassiliev measure at tau filaments as a whole and in parts to rigorously quantify their structural differences (see “Methods” section for definitions).

The linking number is a real number that measures the interwinding of two curves around each other^31^ and can have both positive and negative real values depending on orientations of the curves. The writhe is a measure of self-entanglement of one curve and can have both positive and negative values depending on the orientation of the curve (which we can interpret as handedness of a structure). The writhe is strongly affected by local geometrical complexity. In particular in proteins, high writhe values may not necessarily reflect the topological complexity of the proteins, as they are significantly affected by the presence of secondary structure elements^26^. Topological and geometrical complexity of a curve in 3-space can be decoupled by using the second Vassiliev measure^29^. In general, the second Vassiliev measure indicates the higher order complexity of three-dimensional conformations that capture aspects related to potential knotting. In the context of this manuscript, very small values of the second Vassiliev measure, indicate absence of knotting, but presence of a topological characterization known as knotoids, which are, related to knots. For examples of the linking number, writhe and second Vassiliev measure, see Fig. 12.

All these topological parameters can be applied to a tau filament as a whole or to parts of it (we will also say fragments). In the following we will examine the writhe and second Vassiliev measure of the whole tau filament, which capture its geometrical/topological complexity and the linking number between neighboring stacked filaments. The linking number will also be used within fragments of a tau filament, which will be encoded in a linking matrix.

### Topological classification of tauopathies

In this section we will analyze the mathematical topology and geometry of tauopathies and provide a novel mathematical classification. The following three dimensional crystal structures, available in the Protein Data Bank (PDB)^32^, are used for filaments of tauopathies: *5o3t* for AD (SF), *5o3l* for AD (PHF), *7nrq* for PART, *6nwp* for CTE (type I), *6nwq* for CTE (type II), *6gx5* for PiD, *6tjo* for CBD (type I), *6tjx* for CBD (type II), *7p6d* for AGD (type I), *7p6e* for AGD (type II), *7p65* for PSP, *7p66* for GGT (type I), *7p67* for GGT (type II), *7p68* for GGT (type III), *7p6a* for GPT (type Ia), *7p6b* for GPT (type Ib) and *7p6c* for GPT (type II). We will analyze the topology and geometry of these tauopathy filaments as a whole (we will refer to this as global topology), as well in fragments (we will refer to this as local topology).

#### Global topology of backbone classification

In this section we analyze the global topology of tauopathies using the writhe, the second Vassiliev measure of a filament and the linking number of stacked filaments of each tauopathy (for an example of stacked filaments see Fig. 13).

**Figure 4:**
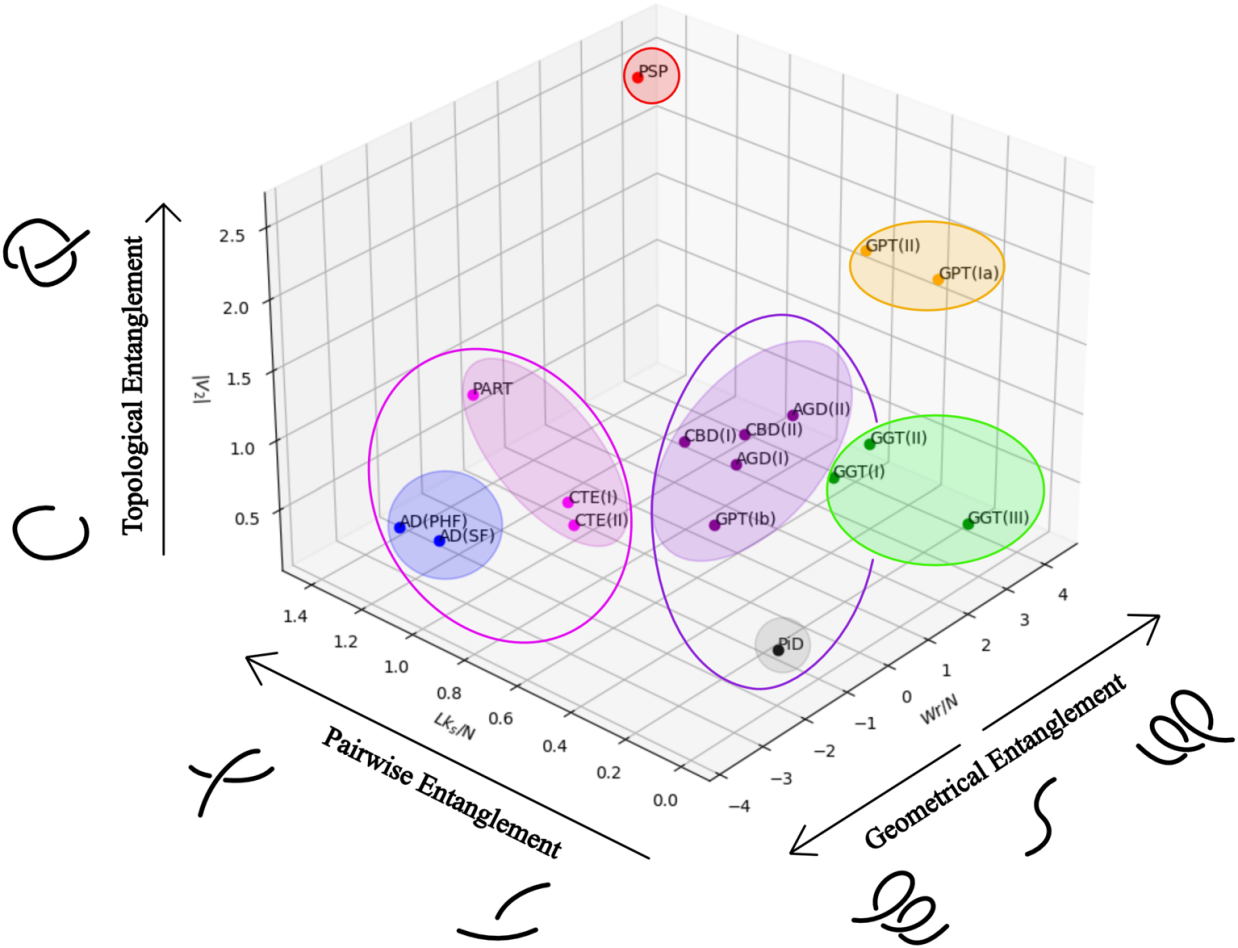
Global topological analysis of tauopathies using the (normalized by length) writhe, *Wr*/*N*, the (normalized) linking number of stacked filaments, $Lk_s/N$, and the absolute second Vassiliev measure, $|V_2|$ (all values are multiples of $10^{-3}$). The data points can be grouped into 7 clusters, shown by the shaded ellipses, with accuracy 0.42. The data can be grouped in 6 clusters (in which case the purple and gray clusters are grouped together), with accuracy 0.42 and in 5 clusters (the magenta and blue clusters are also grouped together) with accuracy 0.40. We find that PSP and, to a lesser extent GPT (type Ia and II), are outliers. The filaments of the data points with the large $Lk_s/N$ (AD, PART, CTE, PSP) are characterized by neurofibrillary tangle pathology.

Resulted topological metrics are summarized in Table 1 and visualized in Fig. 4. All values are multiples of $10^{-3}$. The writhe and the linking number are normalized by the length of a filament to enable for better comparison between filaments, while the second Vassiliev measure is not normalized. The reason is that the writhe and the linking number are affected by local conformations and stored lengths of the filaments, while the second Vassiliev measure is not. Although the obvious flat conformation of filaments and the absence of helical structure elements and knots in filaments result in small values of these mathematical measures, these topological metrics attain non-zero values that reveal subtle differences between tauopathies. As shown by the values of the absolute second Vassiliev measure, PSP and GPT (type Ia and II) have the higher degree of higher order global topological complexity while AD and GGT have a high absolute normalized writhe, which indicates a higher local complexity. AD, PART, CTE and PSP (all of which are characterized by neurofibrillary tangle (NFT) pathology in neurons^33^) have the highest normalized linking number with stacked filaments.

**Table 1:** Global topological metrics of tauopathies. Values are multiples of $10^{-3}$. Each writhe and linking number of stacked filaments are normalized by the length of the corresponding filament.

	$3R + 4R$					3*R*					
Global topology	AD		PART	CTE		PiD					
	SF	PHF		I	II						
*Wr*/*N*	$-3.65$	$-3.59$	$-0.350$	$-0.269$	$-0.104$	$-1.02$					
$Lk_s/N$	0.990	1.15	1.41	1.06	0.904	0.145					
$|V_2|$	0.640	0.585	0.765	0.255	0.360	0.130					

Global topology	4*R*										
	CBD		AGD		PSP	GGT			GPT		
	I	II	I	II		I	II	III	Ia	Ib	II
*Wr*/*N*	$-0.154$	0.603	1.26	0.325	2.79	3.68	4.28	3.68	1.71	0.986	1.15
$Lk_s/N$	0.627	0.518	0.649	0.303	1.26	0.642	0.590	0.138	0.001	0.688	0. 174
$|V_2|$	1.05	1.06	0.620	1.43	2.69	0.105	0.300	0.205	2.39	0.150	2.54

Using the K-Means clustering method^34,35^ on this topological data, filaments are classified into 5, 6, and 7 different clusters shown in Fig. 4 (with accuracy 0.4, 0.42 and 0.42, respectively). The first 5 clusters are (1) AD, PART and CTE; (2) PiD, CBD, AGD and GPT (type Ib); (3) GGT; (4) PSP; (5) GPT (type Ia and II). We notice the different classification of GPT (type Ia) from GPT (type Ib), which suggests global conformational differences between them. The different classification of PSP, GPT and GGT also suggests some global differences in their topology and geometry. These topological differences may reflect distinct pathological features, such as tufted astrocytes in PSP and globular astrocytic inclusions in GGT^33,36,37^. It is also notable that PSP and GGT differ in their associated clinical syndromes, with PSP showing more association with Richardson syndrome and GGT showing more association with semantic variant PPA (svPPA)^38^. The different classification between CBD and AD and PSP may reflect the presence or absence of other regions, like N2, which may play a role in the formation of the different tau aggregates present in different tauopathies^39^. When the number of clusters is increased to 6, the 3R+4R tauopathies are split into two clusters, one with AD and another with PART and CTE . This result indicates different global topology between AD and PART although AD and PART share the same AD fold and suggests that global topology of PART is more similar to CTE. This topological difference between AD and PART may reflect their pathological differences, since PART is characterized by AD-like NFTs without amyloid plaques, which is the pathological hallmark of AD^33,36^. We also note that AD and PART differ in their associated clinical syndromes, with AD being mostly associated with amnestic syndrome, while PART is mostly asymptomatic^38^. With 7 clusters, filaments are further classified with PiD in one cluster and CBD, AGD and GPT (type Ib) in another.

#### Special case: PSP and GPT filaments

**Figure 5:**
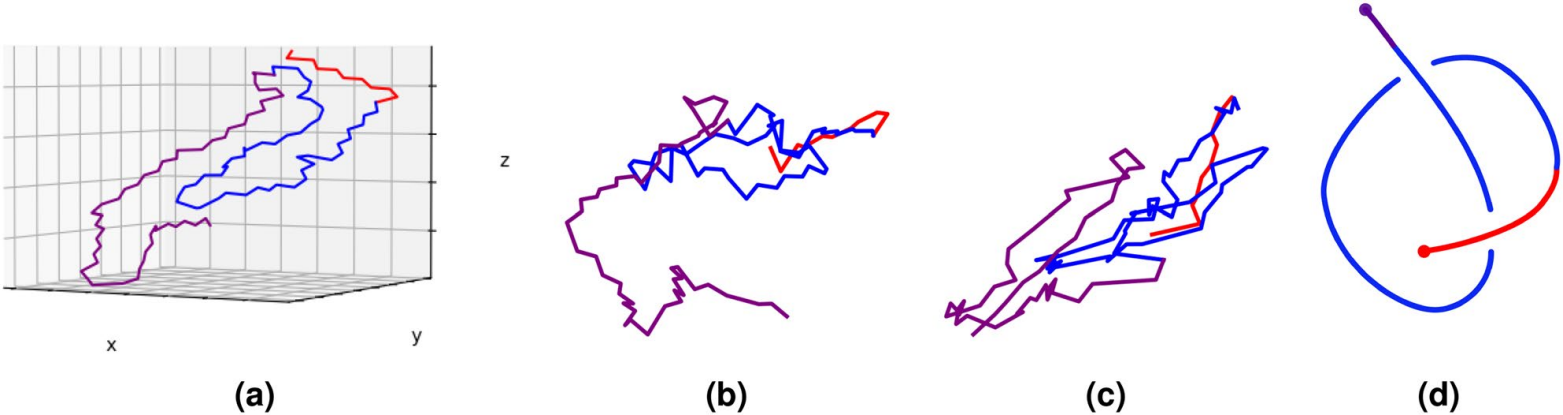
Global topology of the PSP (PDB: *7p65*) and GPT (type II) (PDB: *7p6b*) filaments. The red, blue and purple fragments correspond to residues 272–282, 282–332 and 332–381, respectively. (**a**) A 3D view of the PSP filament. (**b**) A perspective of the PSP filament that indicates complex topology. (**c**) A perspective of the GPT (type II) filament that indicates complex topology. (**d**) The corresponding $K2_1$ knotoid.

In this section we examine the global and local topology of PSP and GPT. The filaments of PSP and GPT (type Ia and II) attain a higher absolute second Vassiliev measure among other filaments, see Table 1, indicating that the structure of these filaments is more complex. The higher second Vassiliev measure of these filaments arises due to its diagrammatic representation of certain projection directions that give a non-zero second Vassiliev invariant (see Methods). That is because, from some points of view, the filaments of PSP and GPT (type Ia and II) are non-trivial knotoids, which is a special, topologically non-trivial, open-ended curve diagram. Indeed, Fig. 5 shows perspectives of the PSP and GPT (type II) filaments that realize the $K2_1$ knotoid structure whose second Vassiliev invariant is equal to 0.5 and which contributes to the higher second Vassiliev measure of these filaments^23^. The red fragment corresponds to residues 272–282 which are three residues at the end of *R*1 repeat and eight residues at the beginning of *R*2 repeat. The blue fragment corresponds to residues 282–332 which are the most of *R*2 and *R*3 repeats. The purple fragment corresponds to the rest of residues in each filament. A weaker signature of this same knotoid structure is observed in the other tauopathies.

#### Local topological classification

**Figure 6:**
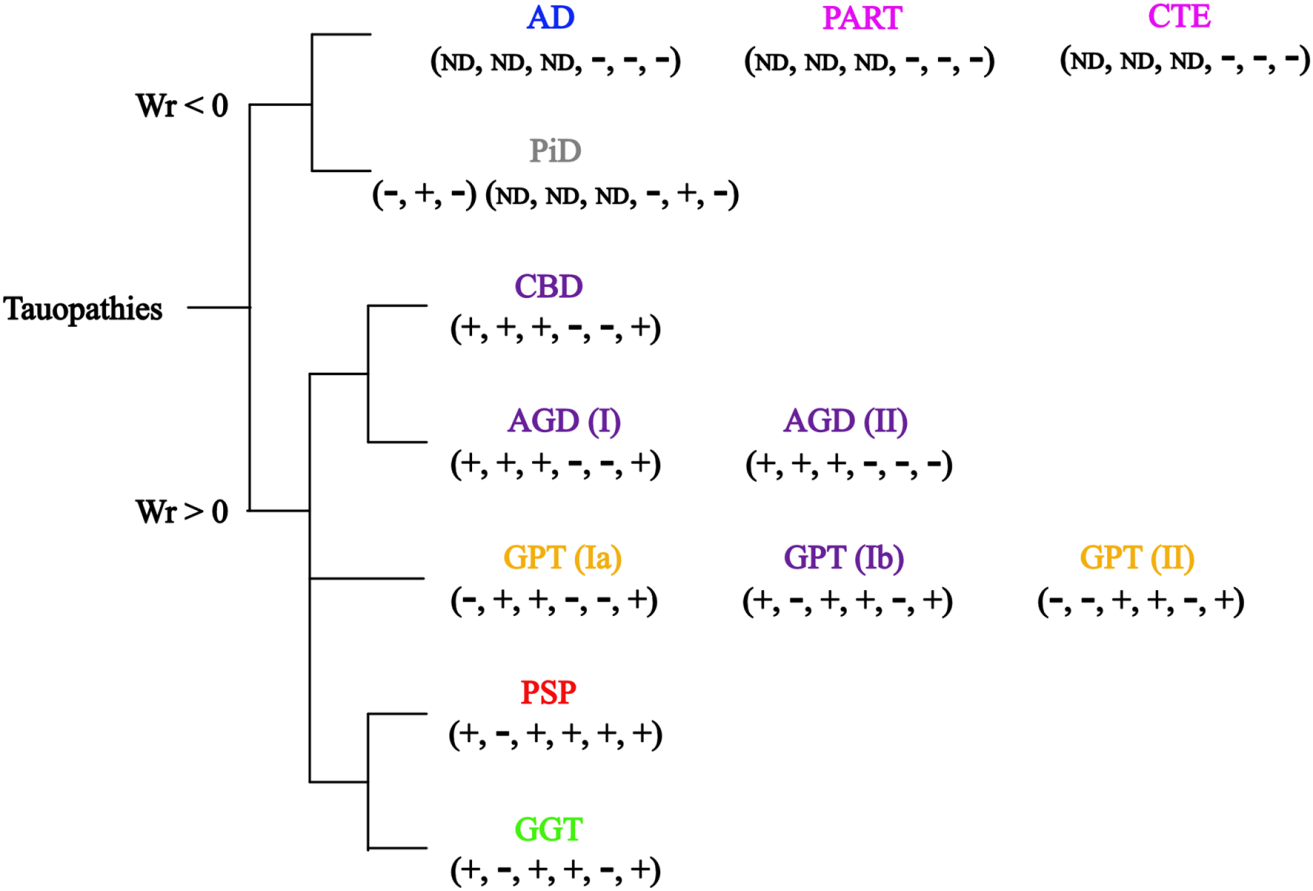
Topological classification of tauopathies based on handedness of global and local structure, as captured by the signs of the writhe of the filaments and the signs of the linking number between repeats within each filament. The signs of linking number between pairs of repeats and pairs of repeats and the C-terminal region are encoded as a 6-tuple of signs, $(\sigma _{lk}(R2, R3), \sigma _{lk}(R2, R4), \sigma _{lk}(R2, C), \sigma _{lk}(R3, R4), \sigma _{lk}(R3, C), \sigma _{lk}(R4, C))$ where the signs of linking number is defined as $\sigma _{lk}$. PiD has an additional 3-tuple $(\sigma _{lk}(R1,R3),\sigma _{lk}(R1,R4),\sigma _{lk}(R_1,C))$. The colors refer to their global topology and geometry classification in 7 clusters in the previous section (see Fig. 4).

In this section, we introduce a topological classification of tauopathies according to the writhe and linking number of fragments of tauopathy filaments. The results are summarized in Fig. 6.

The first level of topological classification of tauopathies comes from the sign of their writhe values which is indicative of handedness of a conformation. The negative writhe values for AD, PART, CTE and PiD are indicative of left-handed conformations, while the positive signs for CBD, AGD, PSP and GGT are indicative of right-handed conformations. (We point out that the two types of CBD differ in sign of writhe, but the negative sign writhe for Type I, has a small absolute value). The handedness may be associated with the preferential cellular localization of tau lesions, since those that are left-handed are mostly neuronal predominant, while the right-handed conformations are neuronal and glial, or glial predominant^33,36,40^. Furthermore, handedness seems to be associated with the absence or presence of neurofibrillary tangles in neurons^33,40^.

In order to understand the role of specific repeats in the structural complexity of tau filaments, we compute their linking numbers. The values of linking number between each pair of repeats or pair of repeats and the C-terminal, for all tauopathies are shown in Table 2. Each value is a multiple of $10^{-2}$. The maximum absolute linking number occurs between R3 and R4 or R4 and C-terminal region for the 3R+4R tauopathies. For the 3R tauopathies, the maximum absolute linking number occurs between R1 and R4, and for the 4R tauopathies, it occurs between R2 and R3 or R2 and R4, with the exception of PSP, for which the maximum linking number is that of R3 and R4 (which is also the maximum value among all pairs in all tauopathies). Although most of the absolute maximum linking number for filaments are attained by pairs of successive repeats/the C-terminal region, the maximum absolute linking number of PiD and AGD occur between pairs of nonsuccessive regions such as between R2 and R4 repeats. Since R3 repeat is involved in most maximum absolute linking numbers, it must attain a conformation that contributes as such, possibly by lying out of the otherwise seemingly planar structure formed by the other repeats.

**Table 2:** Linking number between R1-R4 repeats and between the repeats and the C-terminal region. Values are multiples of $10^{-2}$. “ND” indicates “Not Defined”.

	$3R + 4R$					3*R*					
lk between repeats	AD		PART	CTE		PiD					
	SF	PHF		I	II						
*Lk*(*R*1, *R*3)	ND	ND	ND	ND	ND	$-0.446$					
*Lk*(*R*1, *R*4)	ND	ND	ND	ND	ND	4.55					
*Lk*(*R*1, *C*)	ND	ND	ND	ND	ND	$-1.21$					
*Lk*(*R*2, *R*3)	ND	ND	ND	ND	ND	ND					
*Lk*(*R*2, *R*4)	ND	ND	ND	ND	ND	ND					
*Lk*(*R*2, *C*)	ND	ND	ND	ND	ND	ND					
*Lk*(*R*3, *R*4)	$-2.01$	$-2.85$	$-1.76$	$-1.88$	$-2.80$	$-0.0567$					
*Lk*(*R*3, *C*)	$-1.87$	$-1.89$	$-0.841$	$-1.61$	$-0.590$	0.0720					
*Lk*(*R*4, *C*)	$-2.19$	$-1.59$	$-1.80$	$-1.69$	$-1.82$	$-0.350$					

lk between repeats	4*R*										
	CBD		AGD		PSP	GGT			GPT		
	I	II	I	II		I	II	III	Ia	Ib	II
*Lk*(*R*1, *R*3)	ND	ND	ND	ND	ND	ND	ND	ND	ND	ND	ND
*Lk*(*R*1, *R*4)	ND	ND	ND	ND	ND	ND	ND	ND	ND	ND	ND
*Lk*(*R*1, *C*)	ND	ND	ND	ND	ND	ND	ND	ND	ND	ND	ND
*Lk*(*R*2, *R*3)	3.85	4.08	3.64	3.45	0.378	9.88	9.90	8.98	$-1.90$	2.34	$-3.43$
*Lk*(*R*2, *R*4)	3.53	3.32	3.16	3.68	$-4.55$	$-4.75$	$-5.28$	$-4.57$	1.18	$-9.54$	$-6.28$
*Lk*(*R*2, *C*)	1.39	1.72	4.18	0.122	2.06	0.861	0.962	0.843	1.24	2.41	2.02
*Lk*(*R*3, *R*4)	$-0.807$	$-0.991$	$-0.620$	$-0.496$	14.0	4.80	5.36	5.39	$-1.32$	2.88	3.19
*Lk*(*R*3, *C*)	$-0.384$	$-0.465$	$-0.595$	$-0.545$	0.413	$-0.958$	$-1.06$	$-1.07$	$-0.278$	$-1.37$	$-1.42$
*Lk*(*R*4, *C*)	0.352	0.725	0.0262	$-0.0313$	5.24	0.300	0.213	0.255	0.638	0.608	0.685

The signs of the linking number between pairs of the repeats or pairs of repeats and the C-terminal region provide the second and third levels of topological classification. This classification based on the handedness of specific repeats agrees with the isoform composition of tau inclusions (3R + 4R tauopathy and 3R tauopathy) and four-layered (CBD and AGD) and three-layered (PSP, GGT and GPT) folds^2^, but it also distinguishes GPT from PSP. We denote by $\sigma _{lk}(Ri,Rj)$ the sign of the linking number between repeat *Ri* and *Rj*. The signs of linking number between these pairs are encoded in a six-tuple, ($\sigma _{lk}(R2, R3)$, $\sigma _{lk}(R2, R4)$, $\sigma _{lk}(R2, C)$, $\sigma _{lk}(R3, R4)$, $\sigma _{lk}(R3, C)$ and $\sigma _{lk}(R4, C)$). PiD has an additional three-tuple for R1 repeat ($\sigma _{lk}(R1, R3)$, $\sigma _{lk}(R1, R4)$, $\sigma _{lk}(R1, C)$). Within tauopathies with negative writhe, $\sigma _{lk}(R3, C)$ distinguishes PiD from AD, PART and CTE. Within tauopathies with positive writhe, $\sigma _{lk}(R2, R4)$ distinguishes CBD and AGD from PSP and GGT and $\sigma _{lk}(R3, C)$ further distinguishes PSP and GGT. $\sigma _{lk}(R4, C)$ distinguishes AGD Type I (being identical to that of CBD) from Type II. We can quantify and summarize this difference by counting the number of entries in those tuples that are different among tauopathies. We find no difference between AD, PART, CTE and one difference in PiD from those. The right handed tauopathies have different tuples and among them CBD and AGD differ by at most one entry, while CBD and AGD differ by at least 2 entries from PSP, GGT and GPT. PSP differs from GGT by one entry, and PSP and GGT differ from GPT by at least one entry. The latter may be related to the presence (in the case of CBD and AGD) or absence (in the case of PSP and GGT) of ballooned neurons^33^.

**Figure 7:**
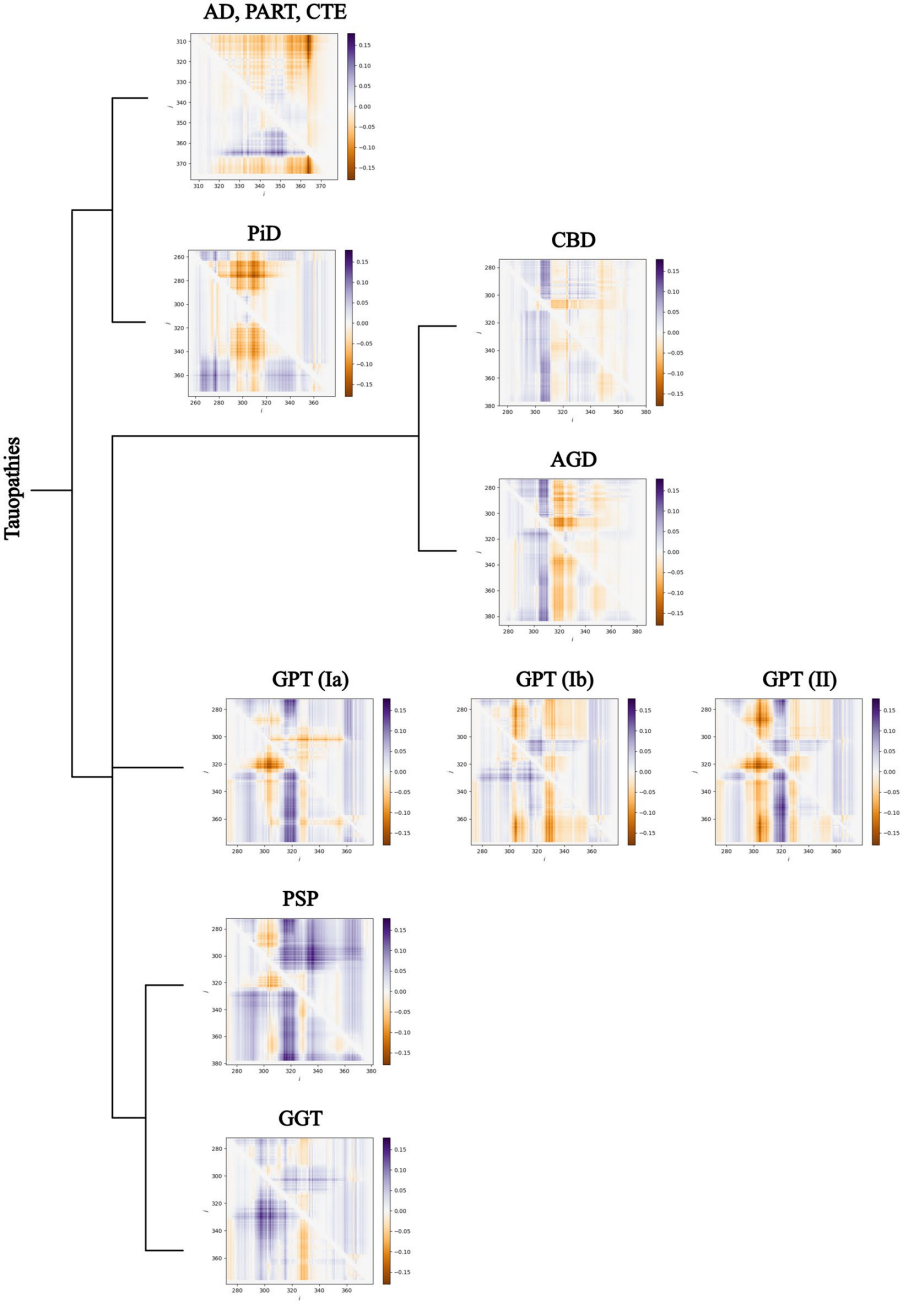
Classification of tauopathies by linking matrices. The linking matrices of AD, PART and CTE are visually similar and not shown here. Also, different filament types within the same tauopathy have visually similar patterns of linking matrices, except of GPT, and are not shown. We identify a vertical band-like pattern in CBD, AGD (x-axis entries 303–310) and, more pronounced, in GPT, PSP, (x-axis entries 315–325) which points to the part of the protein that creates the knotoid motif $k2_1$.

**Figure 8:**
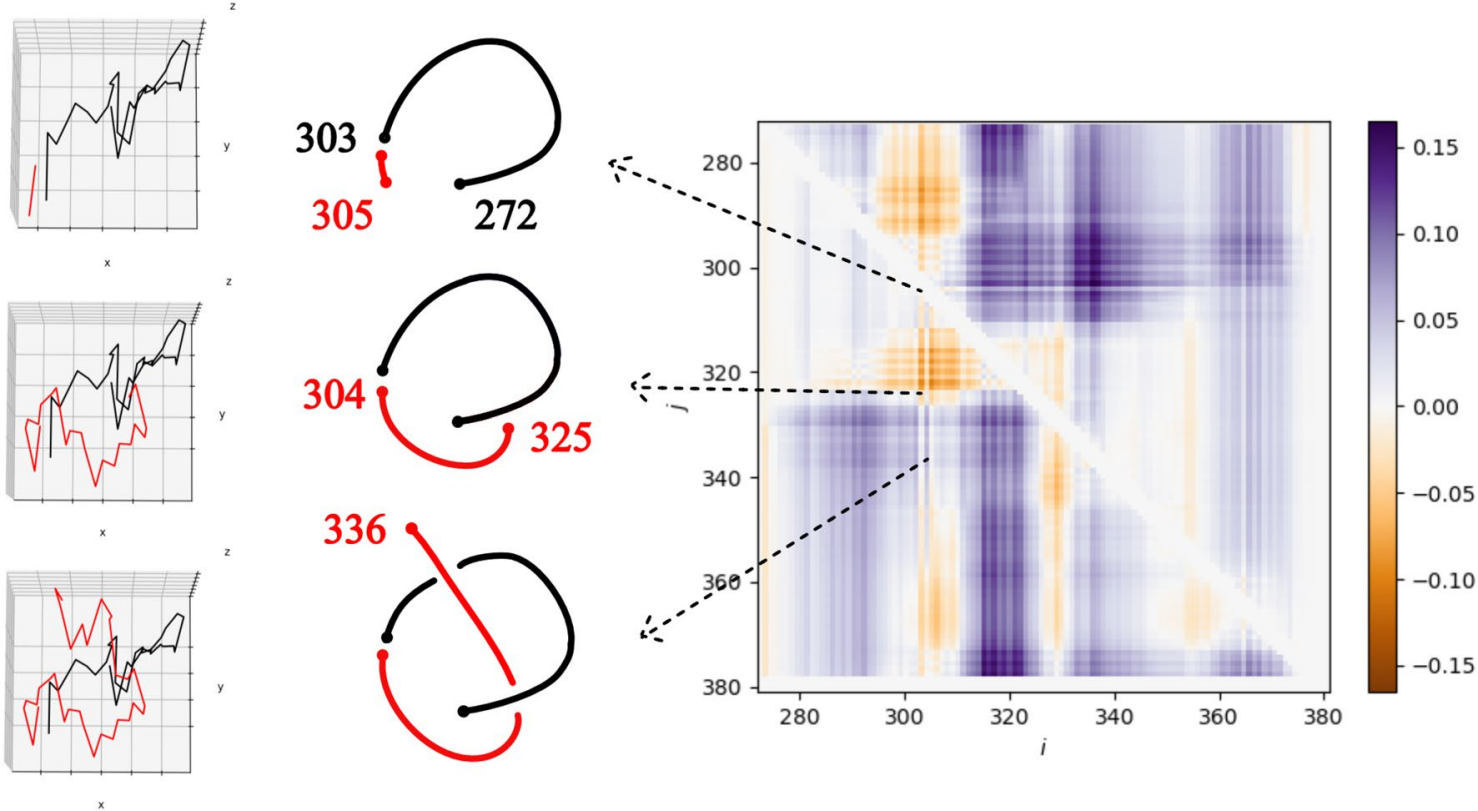
The linking matrix of PSP. Each entry is the linking number between two fragments of the filament. For $i<j$, the entry $a_{i,j}$ is the linking number between the fragment from residue *i* to *j* and the beginning for the filament and $a_{j,i}$ is the linking between the same fragment and the end of the filament. The arrows point to the conformations of fragments of the tau filament, $p_{272, 303}$ (black) and $p_{304, j}$ (red), as the red curve varies for $j = 305, 325, 336$ and their cartoon representations. The linking numbers of these are $-0.0287$, $-0.0375$ and 0.00945, respectively.

A more refined analysis of local pairwise structural complexity can be obtained by the linking matrix^41^. An entry below (resp. above) the diagonal is colored by according to the sign and absolute value of the linking number between the corresponding index fragment of the filament and the preceeding (resp. following) fragment (for a detailed definition, see “Methods”). The linking matrices of tauopathies are shown in Fig. 7. Even though not identical, AD, PART and CTE are very similar and only one of those is shown. Similarly, only one of the different types of CBD, AGD and GGT is shown. By visual inspection we notice that the linking matrices agree with the classification obtained by examining signs of linking number of repeats, but they also reveal more subtle differences. For example, we notice several patterns that are in common between CBD, AGD, GPT and PSP, which are all 4R tauopathies. One feature that is more pronounced is a vertical blue band structure to the right of a diagonal orange region below the diagonal. This is more evident in PSP, shown enlarged in Fig. 8. The orange region is located around the entries approximately (y-axis, x-axis) $= (320,310)$ of the matrix and the blue stripe is located within the *x*-axis entries between 315 and 325, in the beginning of the R3 repeat. As shown in the figure, this pattern captures the presence of a loop-like structure formed by the filament that results in the observed knotoid, discussed in the previous section. Thus, the matrix indicates that this topology includes the PHF6 motif (306–311) in PSP^42–44^. The pattern occurs at a similar location for GPT. CBD and AGD have a weaker signature of this pattern, which is in agreement with their lower values of the second Vassiliev measure. The pattern in CBD and AGD is more narrow and shifted to *x*-axis 303–310, at the interface of the R2 and R3 repeats, which also includes part of the PHF6 motif. These results point to possible connections between topology and geometry of tau filaments and specific repeats and sites therein. The common knotoid pattern may also be associated to the common genetic characteristics of the MAPT H1 haplotype observed in genetic studies of patients of CBD, AGD and PSP^45–47^.

### Side-chain orientation relative classification of tauopathies

**Figure 9:**
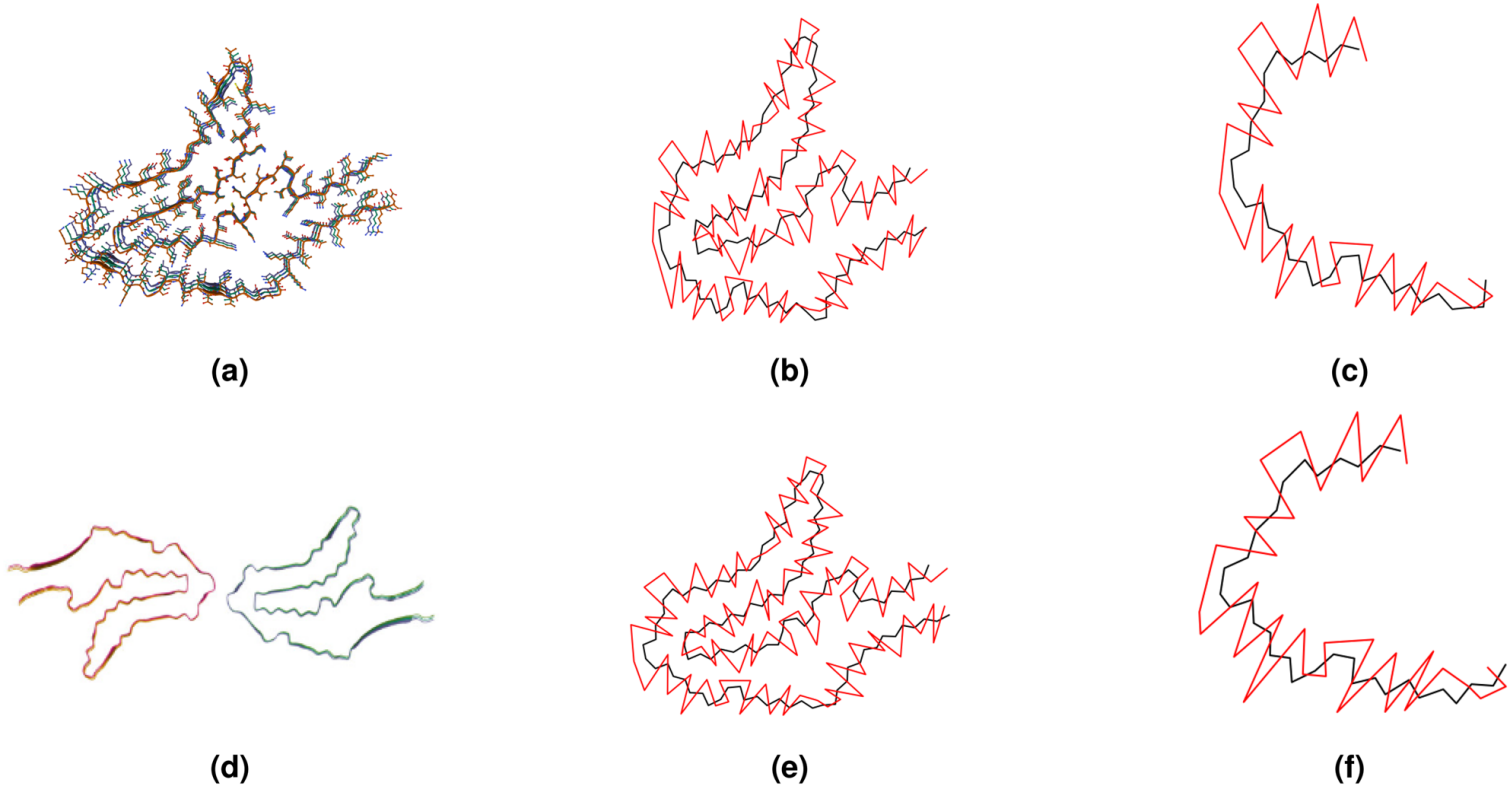
Filaments of CBD (type I and II) represented by their CA backbones (black) and its push-off formed by selected atoms of each residue (red). The relative topology of CA backbone and push-off depends on filament type. The CA backbone and its push-off can also be thought of as forming a ribbon whose twist can be approximated by their linking number. Figures (**a**), (**b**), (**d**), (**e**) and Figures (**c**), (**f**) consist of residues 274–380 and 337–368, respectively. (**a**) The filament of CBD type I (PDB: *6tjo*), consisting of a single protofilament. (**b**) The CA backbone and push-off of the CBD type I filament. Their linking number is $Lk=-5.77$. (**c**) The CA backbone and push-off of the R4 repeat of CBD type I filament. The linking number is $lk_{R4}=-2.46$. (**d**) The CBD type II filament (PDB: *6tjx*), consists of a pair of identical protofilaments. (**e**) The CA backbone and push-off of the CBD type II filament. Their linking number is $Lk=-3.78$. (**f**) The CA backbone and push-off of the R4 repeat of CBD type II filament. The linking number is $lk_{R4}=0.413$, showing a big change to a more right-handed conformation, compared to the same repeat in type I.

So far in this paper, we have examined the filaments of tauopathies as linear open-ended polygonal curves in 3-space constructed from their backbones of CA atoms. The relative position of side-chains with respect to the CA backbone can vary along the backbone. To capture the orientation of side-chains in a way that accounts for the topology/geometry of the backbone, we consider a small push-off of the CA backbone in the direction of the side-chains, which creates an open ribbon-like structure with boundaries, the CA backbone and its push-off. The push-off of a protein CA backbone is constructed from a series of atoms in the side-chains (the R groups) (see Fig. 9). For each side-chain, we choose the non-hydrogen atom in the R group that has the largest distance from the corresponding CA atom. In this section, we analyze the linking number between the CA backbone and its push-off of each tauopathy. We will see that this can also be thought of as the total twist of the ribbon-like structure, thus, the twist of the side-chains around the backbone. To account for the fact that the choice of atom in the R-group may affect the numerical results, we repeat our analysis for other choices, and we only discuss results that are consistent in multiple choices and are therefore independent of the choice of amino acid atom for the push-off.

The linking number between each filament’s CA backbone and its push-off is shown in Table 3 (see also Fig. 9). By comparing these values to the writhe values of the CA backbone, we notice that the linking number of the CA backbone with its push-off is much larger. Thus, by applying the relation of $Tw=Lk-Wr$ for a ribbon, we see that the linking number reported is an approximation of the twist of the push-off^48,49^. CBD type I has the highest absolute linking number. The three-layered 4R tauopathies have right-handed twist (indicated by positive linking numbers), as well as PART and PiD. On the other hand, the four-layered 4*R* tauopathies attain a left-handed twist. Therefore, the relative positions of push-offs are likely to be related to the type of fold seen in a filament.

**Table 3:** The linking number, *Lk* between a CA backbone and a push-off of each tauopathy (and its normalized value by the length of the filament). The normalized values are multiples of $10^{-2}$.

	$3R + 4R$					3*R*					
Global topology	AD		PART	CTE		PiD					
	SF	PHF		I	II						
*Lk*	$-1.96$	1.71	1.93	0.081	1.082	4.60					
*Lk*/*N*	$-2.69$	2.35	2.51	0.108	1.44	4.89					

Global topology	4*R*										
	CBD		AGD		PSP	GGT			GPT		
	I	II	I	II		I	II	III	Ia	Ib	II
*Lk*	$-5.77$	$-3.78$	$-2.67$	$-1.51$	1.70	5.08	4.59	2.38	5.06	3.07	2.56
*Lk*/*N*	$-5.39$	$-3.53$	$-2.32$	$-1.44$	1.55	4.71	4.25	2.21	4.69	2.84	2.37

**Figure 10:**
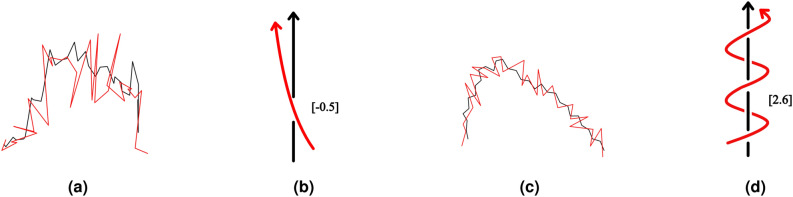
R3 repeat of AD SF and PHF represented by the CA backbone (black) and by a push-off (red) obtained by selected atoms in residues. Each fragment consists of residues 306 - 336. (**a**) R3 repeat of AD (SF) (PDB: *5o3t*). The linking number between the CA backbone and push-off is $Lk=-0.5$. (**b**) A value of linking number $-0.5$ indicates a topological equivalence to a half left-handed turn of the push-off around the CA backbone. (**c**) R3 repeat of AD (PHF) (PDB: *5o3l*). The linking number is $Lk=2.6$. (**d**) A value of linking number 2.6 indicates a topological equivalence to approximately 3 right-handed turns of the push-off around the CA backbone.

Next, we examine the linking numbers of CA backbones and their push-offs in different repeats and the C-terminal region, see Table 4 (see Fig. 10). We find that R3 repeat is the one that shows the largest difference in AD from SF to PHF. In CTE, both R3 and R4 are found to change significantly from type I to type II. This shows that the R3 repeat, which is in contact in the paired filaments, is the one where most backbone and push-off twists change. The R4 repeat is the one that comes in contact in the 4R tauopathies. This is where CBD and GPT shows an increase in right-handed turns from type I to type II. An increase in right-handed turns is also observed for the C-terminal of AGD from Type I to Type II. In GGT we observe a significant increase of right-handed turns around the backbone in the C-terminal from Type I to Type II, III and a significant increase in left-handed turns in the R4 repeat from Type I, II to Type III.

**Table 4:** The linking number between the repeats and C-terminal of CA backbones and push-offs of tauopathies and their normalized values by the length of the repeat. The normalized linking numbers are normalized by a filament length, and the normalized values are multiples of $10^{-2}$.

	$3R + 4R$					3*R*					
Global topology	AD		PART	CTE		PiD					
	SF	PHF		I	II						
$lk_{R1}$	0	0	0	0	0	0.494					
$lk_{R1}/N$	0	0	0	0	0	2.35					
$lk_{R2}$	0	0	0	0	0	0					
$lk_{R2}/N$	0	0	0	0	0	0					
$lk_{R3}$	$-0.529$	2.55	1.46	0.399	3.69	1.92					
$lk_{R3}/N$	$-1.71$	8.23	4.72	1.29	11.9	6.19					
$lk_{R4}$	0.670	1.21	0.149	0.057	$-0.835$	1.50					
$lk_{R4}/N$	2.09	3.77	0.466	0.178	$-2.61$	4.70					
$lk_{C}$	$-1.53$	$-1.15$	0.436	$-0.852$	$-1.03$	0.422					
$lk_{C}/N$	$-15.3$	$-11.5$	3.63	$-7.74$	$-9.32$	4.22					

Global topology	4*R*										
	CBD		AGD		PSP	GGT			GPT		
	I	II	I	II		I	II	III	Ia	Ib	II
$lk_{R1}$	0	0	0	0	0	0	0	0	0	0	0
$lk_{R1}/N$	0	0	0	0	0	0	0	0	0	0	0
$lk_{R2}$	$-1.35$	$-1.35$	0.698	0.200	1.81	1.46	1.06	1.36	1.23	2.12	0.88
$lk_{R2}/N$	$-4.35$	$-4.36$	2.25	0.644	5.84	4.70	3.43	4.38	3.98	6.83	2.84
$lk_{R3}$	$-1.58$	$-1.58$	0.392	$-0.602$	2.34	2.29	1.24	2.25	2.70	1.92	1.96
$lk_{R3}/N$	$-5.11$	$-5.10$	1.26	$-1.94$	7.55	7.40	3.99	7.25	8.72	6.18	6.34
$lk_{R4}$	$-2.46$	0.413	$-1.30$	$-1.39$	$-2.62$	0.302	$-0.058$	$-3.09$	$-1.01$	$-3.34$	0.918
$lk_{R4}/N$	$-7.68$	1.29	$-4.07$	$-4.78$	$-8.18$	0.942	$-0.183$	$-9.65$	$-3.15$	$-10.4$	2.87
$lk_{C}$	$-0.809$	$-0.964$	$-2.78$	$-0.127$	$-0.426$	$-0.049$	1.48	1.41	0.595	0.50	$-0.589$
$lk_{C}/N$	$-6.74$	$-8.03$	$-14.6$	$-0.978$	$-3.27$	$-0.449$	13.5	12.8	5.41	4.59	$-5.35$

These results confirm that the push-off and CA backbone relative topology/geometry is specific to the tau aggregate structure and that the repeats in contact contain most of those changes. We also observe a consistent change in handedness that may be related to specific repeats.

## Discussion

Neurodegenerative diseases formed by aggregated tau proteins are diagnosed by distinct morphologies at different length scales. The process by which these form and how different single filament structures arise, aggregate and form distinct morphologies, is not well understood. Experiments employing cryo-electron microscopy capture the different folds, but cannot explain how they form or quantify the degree to which they differ and in what way.

In this manuscript, by employing novel mathematical methods from topology and geometry, we are able to mathematically classify and quantify the complexity of different tau filaments. Our results reveal that handedness/chirality of conformations varies across tauopathies from the level of the entire tau filament, to the relative position of their repeats. This topological classification of tauopathies captures structural characteristics that correlate pathological characteristics. Higher linking number between stacked filaments is associated with neurofibrillary tangle (NFT) pathology in neurons. A combination of geometrical/topological features (global writhe, second Vassiliev measure and linking with stacked filaments) distinguishes PSP, GGT and CBD, which are associated to tufted astrocytes, globular astrocytic inclusions and astrocytic plaques, respectively. Moreover, PSP and GGT differ in their associated clinical syndromes, with PSP showing more association with Richardson syndrome and GGT showing more association with semantic variant PPA (svPPA).

The handedness of the conformations, indicated by the sign of their writhe, appears to correlate with the preferential cellular localization of tau lesions. Left-handed structures correlate with tauopathies that are mostly neuronal predominant, while right-handed conformations correlate with neuronal and glial, or glial predominant tauopathies. A secondary classification related to the handedness of relative orientations of specific repeats in tauopathies, recovers the isoform composition of tau inclusions (3R + 4R tauopathy and 3R tauopathy) and four-layered (CBD and AGD) and three-layered (PSP, GGT and GPT) folds but, in addition, it also distinguishes GPT from PSP. Our results also reveal that the relative orientation of the side-chains with respect to their backbone depends on the tau fold and type of fold and most changes are observed at specific repeats.

We find that AD which is one of the most prevalent tauopathies, is characterized by a left-handed structure (negative writhe) and has among the highest linking numbers between stacked filaments, indicative of high association between stacked filaments. Even though AD and PART are similar structures, their topology/geometry is different. This agrees with the fact that PART is characterized by AD-like NFTs without amyloid plaques, which is the pathological hallmark of AD, as well as with the fact that the two tauopathies may show different clinical symptoms, with AD being mostly associated with amnestic syndrome, while PART being mostly asymptomatic.

The PSP filament emerges as an outlier among tauopathy filaments, by attaining the highest topological signature. We identify a pattern that contributes to the creation of that topology and notice that it is in the beginning of the R3 repeat for PSP and GGT (containing PHF6) and it is at the interface of the R2 and R3 repeats of AGD and CBD. We notice that all of the latter are 4R tauopathies. The common pattern may be reflecting the common genetic characteristics of the MAPT H1 haplotype observed in genetic studies of patients of CBD, AGD and PSP.

Overall, our analysis reveals that topological metrics of structure capture novel, previously unknown aspects of their structure that can help classify them and point to specific patterns and sites of interest. This new mathematical framework for studying tauopathies could be helpful in quantifying aspects of their topological landscape that lead to aggregation.

## Methods

### Measures of topological characterization of tau filaments

We represent proteins by their consecutive alpha carbon atoms (CA atoms) as linear open-ended polygonal curves in 3-space, which we use as approximations of the protein backbones. In this section, we give the definition of the mathematical tools that we use in this manuscript, namely, the Gauss linking integral, the Writhe and the second Vassiliev measure.

The Gauss linking integral is a measure of interwinding of two curves around each other^31^, and it is defined as follows.

#### Definition 1

(Linking Integral) For two disjoint oriented curves $l_1$ and $l_2$ with arc-length parametrizations $\gamma _1$ and $\gamma _2$ respectively, the *linking integral*, *Lk*, is the double integral over $l_1$ and $l_2$:

1 $$\begin{aligned} Lk(l_1, l_2) = \frac{1}{4\pi } \int _{[0,1]} \int _{[0,1]} \frac{(\dot{\gamma _1}(t) \times \dot{\gamma _2}(s)) \cdot (\gamma _1(t) - \gamma _2(s))}{||\gamma _1(t) - \gamma _2(s)||^3} \, dtds \end{aligned}$$

where $\dot{\gamma }$ denotes the derivative of $\gamma$ and where the integral runs over $[0,1] \times [0,1]$, which denotes all $s, t \in [0,1]$.

**Figure 11:**
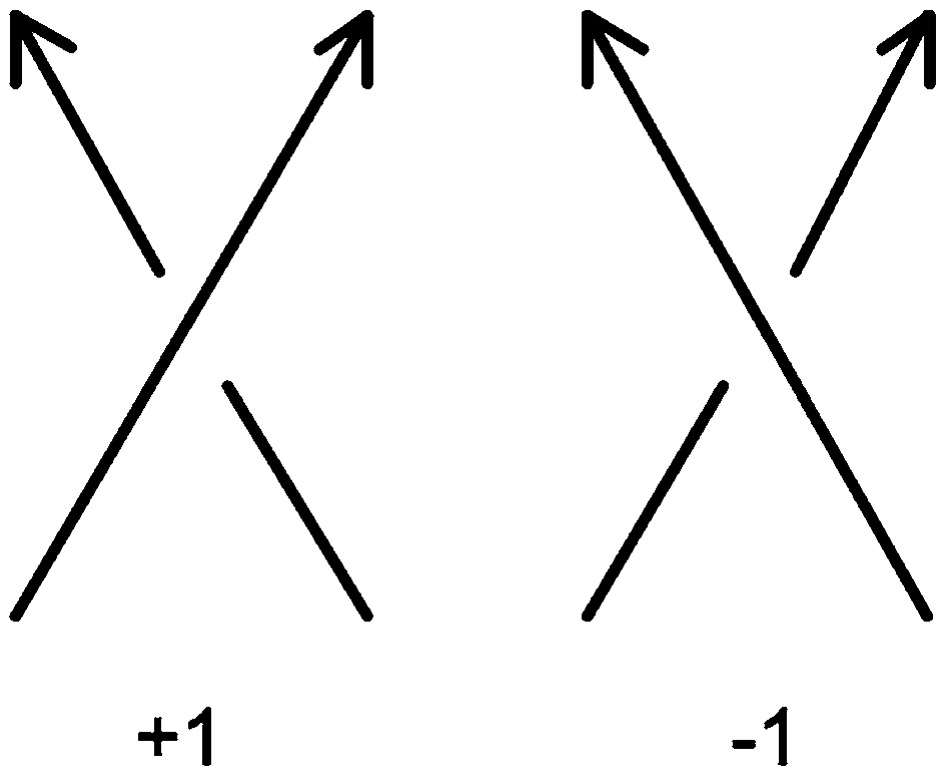
A positive crossing (left) and a negative crossing (right).

The Gauss linking integral is a measure of the number of times two curves wind around and can have both positive and negative values depending on orientations of the curves. The linking integral may be non-zero even for curves that do not visibly interwind. In those cases, it captures aspects of their relative positions related to their orientations and vicinity, which can be interpreted as topological interactions or a potential for interwinding. In this paper, we refer to the linking integral the *linking number* of two curves. The Gauss linking integral can be expressed in terms of properties of link diagrams. An oriented link diagram is a projection of a pair of oriented curves to a plane, where double points keep the information of over/under and each crossing is labeled as a positive crossing ($$+1)$$ or a negative crossing ($$-1$$) based on the relative orientations, see Fig. 11. A positive crossing and a negative crossing are also refereed to as a right-handed crossing and a left-handed crossing, respectively. The linking integral is then the average of half the algebraic sum of signs of all crossings in a projection of two curves over all possible projection directions. For polygonal curves, the linking integral can be expressed as a finite sum of signed geometric probabilities that two edges cross in any projection direction^50^.

The Gauss linking integral can also measure the degree at which a curve interwinds around itself. When applied to one curve, the Gauss linking integral is called the *writhe* of the curve.

#### Definition 2

(Writhe) For an oriented curve *l* with an arc-length parametrization $\gamma$, the *writhe*, *Wr*, is the double integral over *l*:

2 $$\begin{aligned} Wr(l) = \frac{1}{2\pi } \int _{[0,1]^*} \int _{[0,1]^*} \frac{(\dot{\gamma }(t) \times \dot{\gamma }(s)) \cdot (\gamma (t) - \gamma (s))}{||\gamma (t) - \gamma (s)||^3} \, dtds \end{aligned}$$

where $\dot{\gamma }$ denotes the derivative of $\gamma$ and where the integral runs over $[0,1]^* \times [0,1]^*$, which denotes all $s, t \in [0,1]$ such that $s \ne t$.

It is a measure of the number of times a curve winds around itself and can have both positive and negative values depending on an orientation of the curve. Even though a high absolute writhe value may indicate topological complexity, the writhe is sensitive to local geometrical entanglement. The writhe can also be expressed as the average algebraic sum of signs of all crossings in a projection of a curve with itself over all possible projection directions.

Higher order topological entanglement of a curve in 3-space can be decoupled from local geometrical entanglement by using the second Vassiliev measure^29^.

#### Definition 3

Let $j_1< j_2< j_3 < j_4$ be points on a knot or link diagram. We will say that this four-tuple corresponds to an alternating crossing when $j_1, j_3$ and $j_2, j_4$ are two crossing points in the diagram such that if $j_1$ belongs to the over-arc in the crossing, $j_2$ belongs to the under-arc in the crossing or vice-versa.

#### Definition 4

(Second Vassiliev measure) For an oriented curve *l* with the arc-length parametrization $\gamma$, the second Vassiliev measure, $v_2$, is the double alternating self-linking integral over *l*:

3 $$\begin{aligned} v_2(l) = \frac{1}{4\pi } \int _{\xi \in S^2} \frac{1}{2} \sum _{j_1<_2<j_3<j_4 \in I_{\xi }} \epsilon (j_1, j_3) \epsilon (j_2, j_4) \, dA \end{aligned}$$

where $\epsilon (s,t) = \pm 1$ is the sign of the crossing between the projection of $\gamma (s)$ and $\gamma (t)$, and where $I_{\xi }$ denotes the set of pairs of alternating crossings in the projection to the plane with normal vector $\xi$.

The second Vassiliev measure is the average of half algebraic sum of a product of signs of alternating crossings (a certain pattern of crossings) in a projection of a curve over all possible projection directions. In practice, we use 100,000 projections, which we found is sufficient for accurate results for our dataset through a convergence analysis. It is a measure of knottedness and can have both positive and negative values depending on an orientation of a curve. For a polygonal curve, the second Vassiliev measure can be expressed as a finite sum of geometric probabilities related to the complexity of knotoid diagrams (a projection of an open-ended curve onto a plane)^29^. In general, high values of the second Vassiliev measure indicate knotting while very low values, which is the case in this study, indicate the complexity of three-dimensional conformations that could lead to knotting. For a closed curve, the second Vassiliev measure is called the second Vassiliev invariant and it is a topological invariant.

In proteins, high writhe values may not necessarily reflect the topological complexity of the proteins, as they are significantly affected by the presence of secondary structure elements such as helices. The second Vassiliev measure, on the other hand, reflects the topological complexity of the proteins on the basis of knottedness of the proteins. We stress however that the structures of tauopathies analyzed here are not knotted and v2 in that case measures the presence of knotoids, which are perspective-dependent non-trivial topological structures.

**Figure 12:**
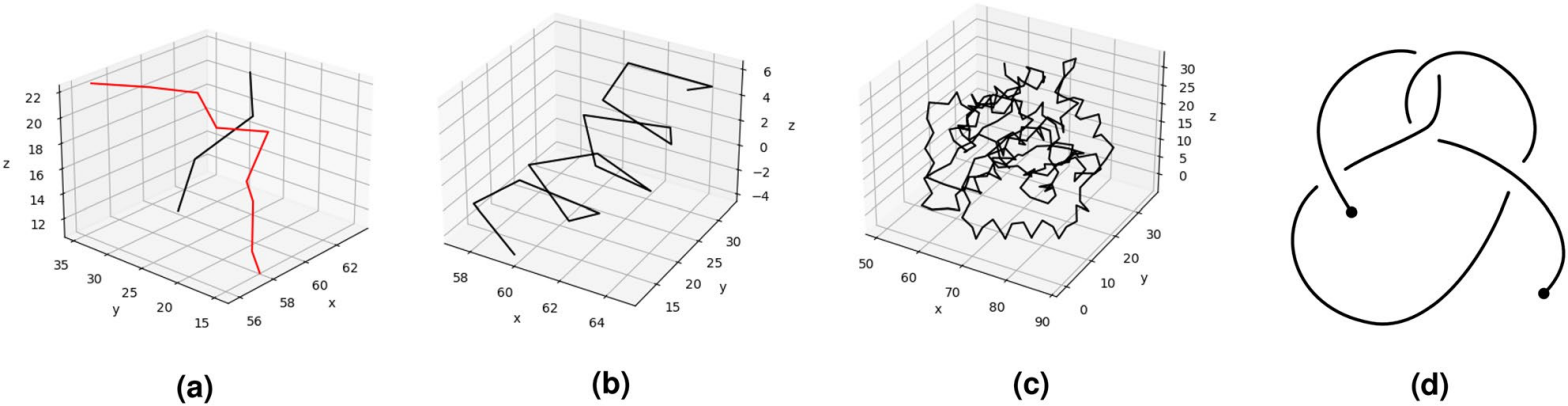
Measures of topological characterization of a knotted protein. The ubiquitin Carboxy-terminal Hydrolase L1 (PDB: *2etl*) is used as an example. (**a**) the black and red curves are parts of the ubiquitin protein, corresponding to residues 26–30 and 215–223, respectively. Their linking number is $Lk \approx -0.22$. (**b**) the writhe and second Vassiliev measure of a helix, residue 192–207, in the ubiquitin protein is $Wr \approx 1.65$, while its second Vassiliev measure is $v_2 = 0.0$. (**c**) The second Vassiliev measure of the full length ubiquitin protein, $v_2 \approx 1.56$, is indicative of knotting. To be compared to the second Vassiliev measure of the knot $5_2$ is $v_2=2$. (**d**) An open $5_2$ knot.

**Figure 13:**
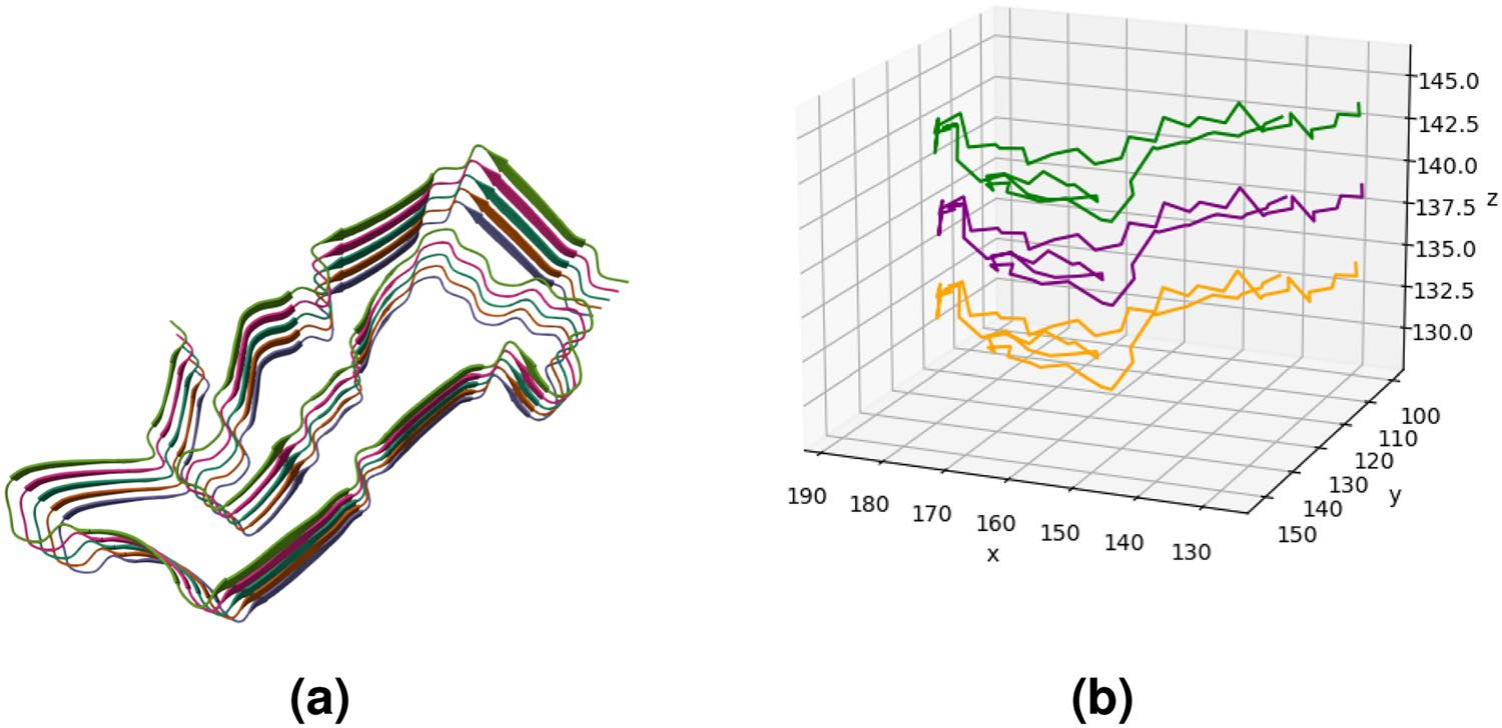
Stacked filaments of PSP (PDB: *7p65*). The residues 272–381 are present in these filaments. (**a**) Each filament in the structure interacts with two others. (**b**) A filament in PSP has normalized writhe $Wr/N \approx 2.8 \times 10^{-3}$ and second Vassiliev measure $|v_2| \approx 2.69 \times 10^{-3}$, which indicate global geometrical and topological complexity. The purple filament is in the immediate vicinity of both the green and the yellow filaments. We assign the linking number with neighboring filaments to the purple filament by averaging its linking number with the green and yellow filaments. The normalized linking number with neighboring filaments for PSP is $Lk_{s}/N \approx 1.26 \times 10^{-3}$.

For open curves in 3-space, like proteins, the Gauss linking integral, the writhe and the second Vassiliev measure, are continuous functions of the curve coordinates. For examples of the linking number, writhe and second Vassiliev measure, see Figs. 12 and 13.

All these topological parameters can be applied to a protein structure as a whole or to fragments of it. In the following we will examine the writhe of the whole tau filament, as well as the linking between repeats within a tau filament, which reflects the complexity of the relative position of repeats within a tau filament. Similarly, one can examine all possible relative positions of parts of the tau filament with the rest of it. That information can be encoding in the linking matrix^41^:

#### Definition 5

(Linking Matrix) Let $p_{k,l}$ denote the part of a protein filament from residue *k* to residue *l* and let the first residue of the protein be *K* and the last *N*. The linking matrix of a filament is the matrix with entries $a_{i,j} = Lk(p_{K,i-1}, p_{i,j})$, and $a_{j,i}=Lk(p_{i,j},p_{j+1,N})$, where $i<j$.

The information contained in the linking matrix can be visualized in the form of a coloured map, called the linking fingerprint of a tau filament. The colors represent the positive and negative linking values and their intensity, the magnitude of those.

## Data Availability

All data generated or analysed during this study are included in this published article.
